# Retrospective study revealed integration of CNV-seq and karyotype analysis is an effective strategy for prenatal diagnosis of chromosomal abnormalities

**DOI:** 10.3389/fgene.2024.1387724

**Published:** 2024-05-23

**Authors:** Yunsheng Ge, Jiayan Chen, Yanru Huang, Di Shao, Wenbo Wang, Meijiao Cai, Meihua Tan, Jian Zhang

**Affiliations:** ^1^ Department of Central Laboratory, Women and Children’s Hospital, School of Medicine, Xiamen University, Xiamen, China; ^2^ BGI Genomics, Shenzhen, China; ^3^ Liangzhu Laboratory, Zhejiang University Medical Center, Hangzhou, China

**Keywords:** chromosomal abnormality, prenatal diagnosis, CNV-seq, karyotype analysis, ultrasonic abnormality

## Abstract

Fetal chromosomal abnormalities are the main cause of adverse pregnancy outcomes and are the focus of invasive prenatal diagnosis. Recent studies have demonstrated that various techniques have distinct advantages. Achieving high-resolution and effective prenatal chromosomal abnormality diagnosis requires a multi-technology integration strategy. Based on retrospective samples from a single center, we propose that integrating CNV-seq and karyotype analysis is an effective strategy for prenatal diagnosis of chromosomal abnormalities. In this study, 13.80% of the pregnant women (347/2514) were found to have likely pathogenic or pathogenic fetal chromosomal abnormalities using this integrated approach. Among these cases, 53.89% (187/347) had consistent chromosomal abnormalities detected by both CNV-seq and karyotyping analysis, while 19.02% (66/347) and 27.09% (94/347) of cases were diagnosed solely by CNV-seq or karyotyping, respectively. Fetal chromosomal abnormalities were identified in 18.39% of samples with abnormal ultrasound, which was significantly higher than the percentage found in samples with normal ultrasound (*p* < 0.001). Samples with multiple ultrasound abnormalities and single-indicator ultrasound abnormalities such as nasal bone dysplasia, renal dysplasia, or echogenic fetal bowel also had higher rates of chromosomal abnormalities (*p* < 0.05) compared to normal samples. Analyzing samples with Trio family data (N = 521) revealed that about 94% of variants of uncertain significance were inherited from parents and were non-pathogenic. Overall, integrating CNV-seq and karyotype analysis is an effective strategy for prenatal diagnosis of chromosomal abnormalities. This study provides valuable insights for correlating prenatal screening indicators with chromosomal abnormalities.

## 1 Introduction

Fetal chromosomal abnormalities are the primary cause of adverse pregnancy outcomes and the main focus of invasive prenatal diagnosis. Recent studies have shown that different techniques have their own advantages. High-resolution and effective prenatal chromosomal abnormality diagnosis relies on a multi-technology integration strategy (Ma et al., 2021). Karyotyping has been the gold standard for identifying chromosomal abnormalities in prenatal diagnosis for over 50 years, with a maximum resolution of 3–5 Mb (Vermeesch et al., 2007). Typically, karyotyping can detect true mosaicism as low as 5% (Hook, 1977; Ma et al., 2021). CNV-seq, specifically low-coverage whole genome sequencing, is widely used in clinical diagnosis due to its simple experimental operation, lack of probe design, and high detection performance. Its sensitivity and specificity range from 72.2% to 96.5% and 91.7%–99.9%, respectively, at different sequencing depth (Xie and Tammi, 2009). Using an integrated strategy of CNV-seq and karyotype analysis, we conducted a comprehensive analysis of prenatal chromosomal abnormality diagnosis for singleton pregnant women at Xiamen Maternity and Child Health Hospital from March 2020 to December 2021.

## 2 Materials and methods

### 2.1 Subjects

Using an integrated strategy of CNV-seq and karyotype analysis, we conducted a comprehensive analysis of prenatal chromosomal abnormality diagnosis for singleton pregnant women at Xiamen Maternity and Child Health Hospital from March 2020 to December 2021. A total of 2514 prospective pregnant women were involved in this study. Samples for fetal chromosomal abnormality diagnosis were obtained from amniotic fluid, chorionic villus, and cord blood samples. Furthermore, 521 out of the 2514 fetal samples were confirmed by Trio family verification. The samples for testing chromosome abnormalities in fetal parents were obtained from peripheral blood. The participants in this study had signed informed consent prior to undergoing chromosome abnormality testing, and also agreed to allow the use of their testing results for scientific research or reporting purposes, provided that their personal information was removed. The Ethics Committee of Xiamen Maternity and Child Health Hospital approved this study (protocol code KY-2023-155-K01).

### 2.2 Clinical information

Basic clinical information of pregnant women was collected prior to undergoing prenatal chromosomal abnormality diagnosis. This information includes age, gestational week, history of adverse pregnancy, and the results of ultrasound diagnosis for structural or soft marker abnormalities. The ultrasound diagnosis results were classified by professionals.

### 2.3 CNV-seq analysis

The processes of detecting fetal chromosomal abnormalities by CNV-seq were consistent with previously published studies (Wang J. et al., 2018; Lan et al., 2021; Tan et al., 2022). Genomic DNA was firstly isolated according to the instructions of a commercial DNA extraction kit for different biological samples. Whole-genome sequencing libraries were then constructed using the MGIEasy universal DNA Library Prep Set (MGITech) and raw sequencing data were generated on the MGISEQ-2000 sequencer platform (MGITech) using a single-end 35bp strategy. The raw reads were aligned to the human genome (hg19) using SOAP2 (Li et al., 2009), and CNVs were then detected using PSCC(Li et al., 2014). Finally, the pathogenicity of CNVs was deciphered using a commercial software provided by BGI Genomics, which follows the ACMG guidelines (Riggs et al., 2020), and is an updated software of AutoCNV, a semiautomatic CNV interpretation system (Fan et al., 2021). In this study, we defined CNVs with likely pathogenic and pathogenic effects as results of chromosomal abnormalities. Furthermore, we conducted genetic origin analysis of VUS variants using Trio pedigree sample data and identified VUS variants inherited from parents as likely benign CNVs.

### 2.4 Karyotype analysis

Ultrasound-guided invasive procedure was carried out at different pregnancy weeks, collecting amniotic fluid, chorionic villi, or umbilical cord blood samples. All of the samples were cultured following the standard protocols. Chromosome preparations were G-banded using trypsin-Giemsa staining for cytogenetic karyotyping after a series of standard protocols including colchicine treatment, hypotonic treatment, fixation and centrifugation. Chromosome karyotype map scanning and acquisition were done using an automatic metaphase chromosome analysis system (Axio Imager Z2, ZEISS IKAROS, Germany). Samples from each pregnant woman were cultured and Karyotype analysis for two lines. At least 40 karyotypes were counted for each case, and five karyotypes were randomly selected for analysis. Karyotypes were diagnosed according to the international system for human cytogenetic nomenclature (ISCN, 2016).

### 2.5 Follow-up and statistical analysis

All pregnant women were followed up to the pregnancy outcomes. Microsoft Excel (version 16.16.27) and R (version 4.1.2) were used for data analysis. Data are reported with the descriptive statistics method and measurement data are expressed as the mean ± SD. A chi-squared test was used to analyze differences among the two groups. *p* < 0.05 was considered statistically significant.

## 3 Results

### 3.1 Clinical characteristics

**FIGURE 1:**
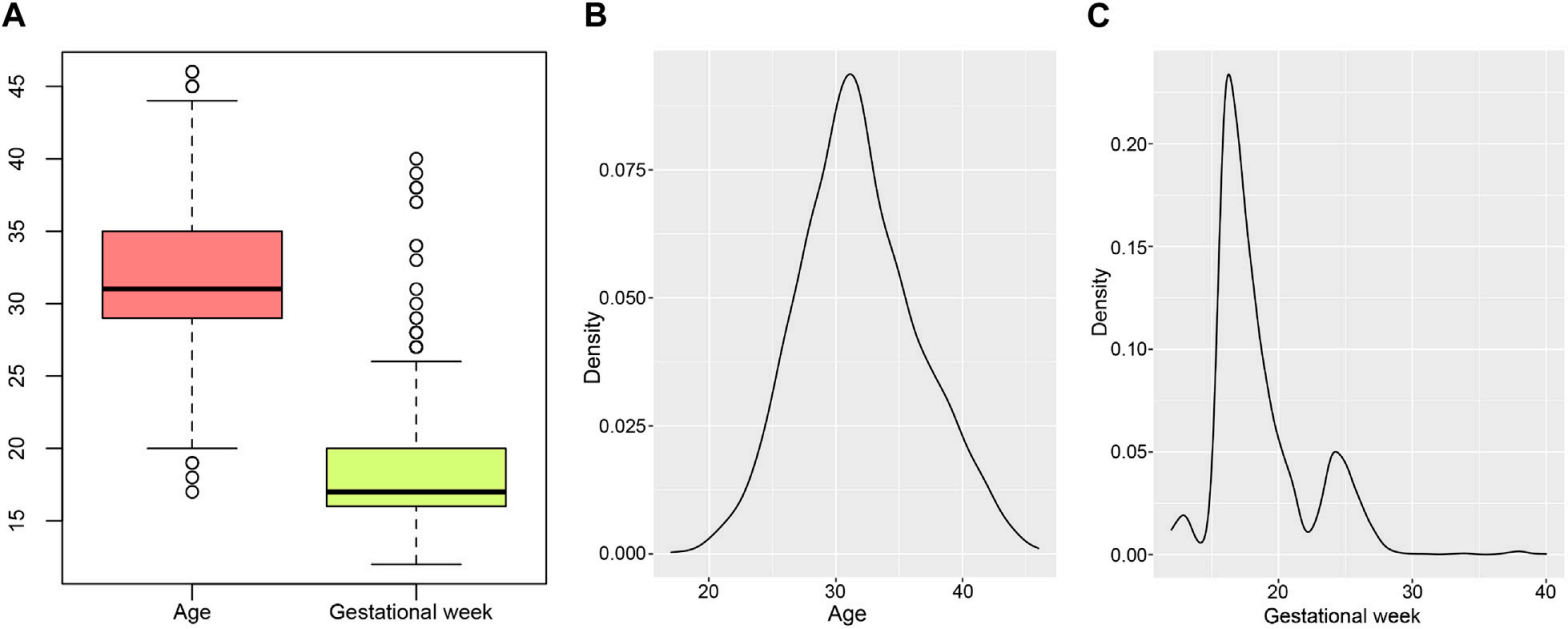
Age and gestational weeks distribution of pregnant women. **(A)** is the boxplot of ages and gestational weeks. **(B, C)** are density curves of age and gestational week separately.

The study included 2514 prospective pregnant women, the mean age was 31.83 ± 4.74 years and the mean gestational week was 18.58 ± 4.74 (Figure 1A). The density curves showed that the peak age was 31.13 years old, and the peak periods for prenatal diagnosis were at 16.27 and 24.25 gestational weeks (Figures 1B, C). Statistics of clinical features were summarized in Table 1. Of the sample population, 27.09% were pregnant women with advanced maternal age (AMA) (age ≥35 years old), while 72.91% were younger than 35 years old. The sample types included amniotic fluid (79.40%, 1996/2514), chorionic villus (4.02%, 101/2514), and cord blood (16.59%, 417/2514). The number of pregnant women with adverse pregnancy history was similar to that of pregnant women without adverse pregnancy history, accounting for 50.95% and 49.05% respectively. Based on the follow-up statistics, 83.77% of pregnant women parturiated, 15.71% of pregnant women underwent iatrogenic abortion, and 0.52% of pregnant women experienced spontaneous abortion.

**TABLE 1:** Summary of clinical characteristics and chromosomal abnormalities.

Characteristics	Number of cases	Percentage (%)	Chromosomal abnormality case	Chromosomal abnormality rate (%)	*p*-Value
Age					
<35	1833	72.91	250	13.64	0.745
≥ 35	681	27.09	97	14.24	
Sample type					
Amniotic fluid	1996	79.40	247	12.37	<2.2E-16
Chorionic villus	101	4.02	48	47.52	
Cord blood	417	16.59	52	12.47	
Adverse pregnancy history					
No	1233	49.05	183	14.84	0.154
Yes	1281	50.95	164	12.80	
Ultrasonic abnormalities					
No	1399	55.65	142	10.15	3.881E-09
Yes	1115	44.35	205	18.39	
Pregnancy outcome					
Spontaneous abortion	13	0.52	1	7.69	1.240E-04
Iatrogenic abortion	395	15.71	195	49.37	
Parturition	2106	83.77	151	7.17	

### 3.2 The integration of CNV-seq and karyotype analysis effectively improved the diagnostic efficiency of chromosomal abnormalities

**FIGURE 2:**
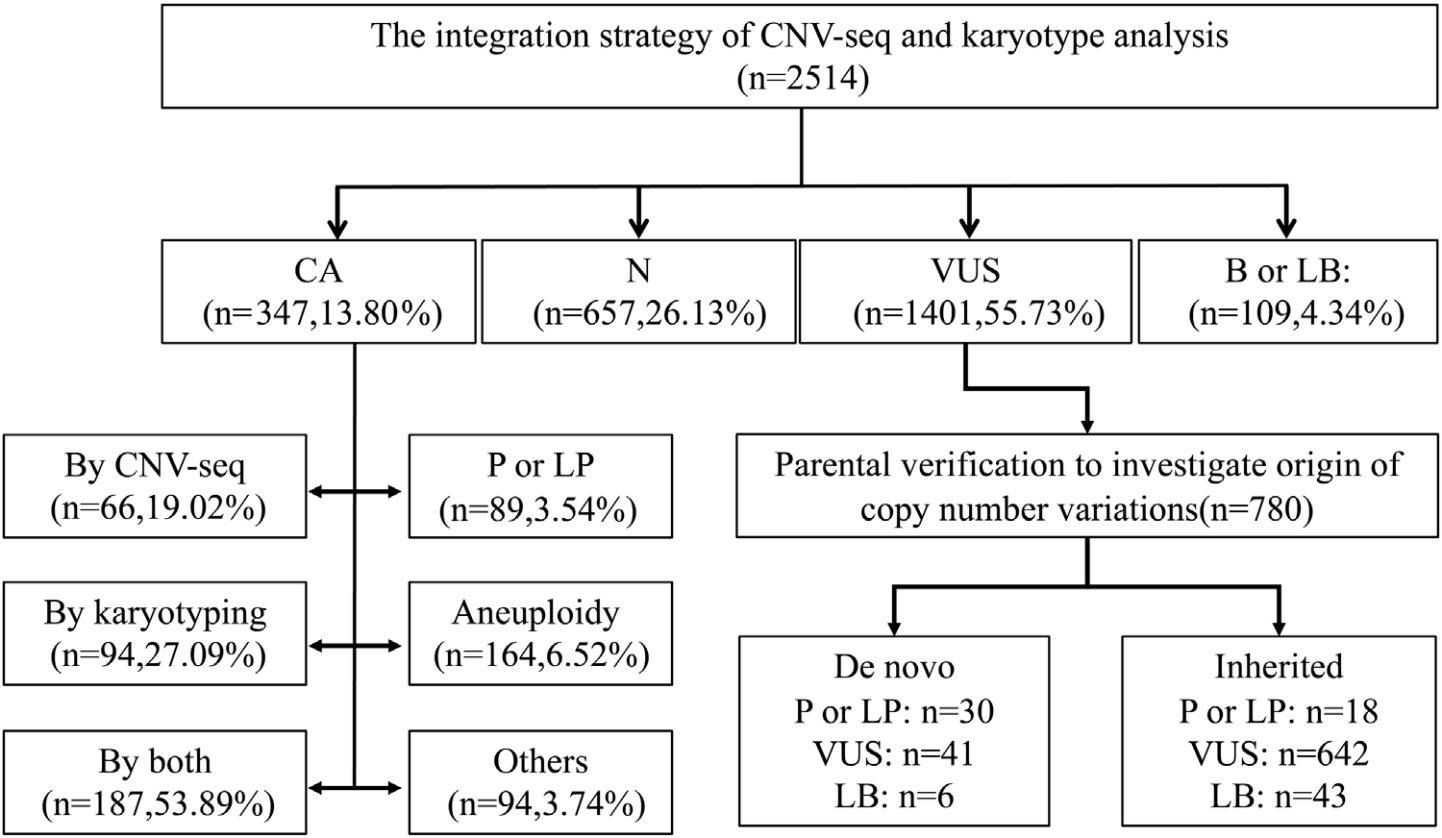
Flowchart of the integration strategy of CNV-seq and karyotype analysis in 2514 prenatal cases. CA, Chromosomal abnormalities. N, Negative. VUS, Variants of Uncertain significant. B or LB, Benign or Likely Benign Variants. P or LP, pathogenic or likely pathogenic CNVs.

**FIGURE 3:**
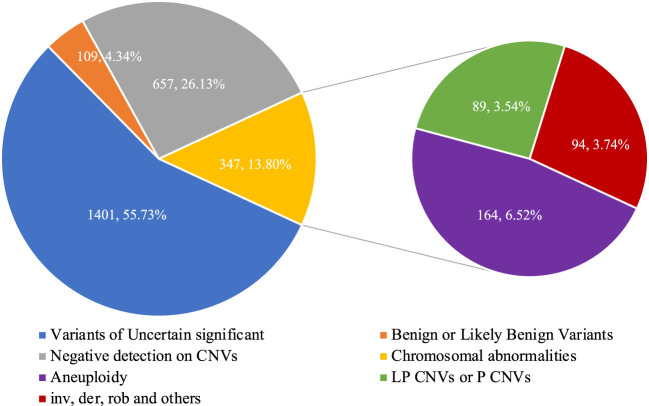
The detection rate of chromosomal abnormalities by integrated analysis.

The flowchart of the integration strategy of CNV-seq and karyotype analysis was demonstrated in Figure 2. By this integration strategy we identified 347 cases of fetal chromosomal abnormalities in 2514 pregnant women undergoing prenatal diagnosis, resulting in a diagnostic rate of chromosomal abnormalities was 13.80% (Figure 2; Supplementary Table S1). In the remaining samples, 1401 (55.73%) fetuses were detected with variants of uncertain significant, 109 (4.34%) fetuses were detected with benign or likely benign variants, and 657 (22.96%) fetuses have no CNVs detected (Figure 3). Out of the 347 samples, 187 (53.89%, 187/347) samples showed consistent results between CNV-seq and karyotyping analysis in terms of chromosomal abnormalities. Meanwhile, 66 (19.02%, 66/347) and 94 (27.09%, 94/347) samples were exclusively diagnosed by CNV-seq and karyotyping analysis, respectively (Supplementary Tables S2, S3). In the 66 samples detected solely by CNV-seq, all exhibited CNVs with sizes ranging from 132.32 Kb to 4.60 Mb, exceeding the scope of karyotyping analysis (Supplementary Table S2). In contrast, the 94 samples detected solely by karyotyping analysis included 25 cases of translocations (16 mutual translocations, 9 Rothschild translocations), 48 inversions, 18 chimeras, 3 chromosomal polymorphisms, and 1 triploid, which also fall outside the detection range of CNV-seq technology (Supplementary Table S3).

The clinical features and its corresponding detection rates of chromosomal abnormalities are shown in Table 1. No statistically significant differences in chromosomal abnormalities were detected between the AMA group and the younger group (Table 1). The proportion of chorionic villus samples was the lowest (4.02%, 101/2514), but the detected rate of chromosomal abnormalities was significantly higher than the other two groups (*p* < 0.001). Based on pregnancy outcomes, it has been found that 49.37% of fetuses resulting from iatrogenic abortions have chromosomal abnormalities. This highlights the importance of fetal chromosome diagnosis in determining appropriate clinical intervention strategies. The spectrum of chromosome aneuploidy and pathogenic or likely pathogenic CNVs were summarized in Table 2. T21 and del (15q11.2) were the most common chromosomal aneuploidies and pathogenic CNVs, with detection rates of 2.74% (69/2514) and 0.36% (9/2514). Among the 98 cases with pathogenic or likely pathogenic CNVs, 6 of them have detected with 2 pathogenic or likely pathogenic CNVs, and 3 of them were detected with 1 pathogenic or likely pathogenic CNVs and 1 aneuploidy (Supplementary Table S2). The population frequency statistics of all chromosomal abnormalities detected by CNV-seq in our cohort were presented in Supplementary Table S4, T21 and T18 are the most common chromosomal abnormalities.

**TABLE 2:** Spectrum of aneuploidy and pathogenic or likely pathogenic CNVs.

Aneuploidy	Number of cases	Detection rate (%)	Pathogenic or likely pathogenic CNVs	Number of cases	Detection rate (%)
T21	69	2.74	del (15q11.2)	9	0.36
T18	28	1.11	del (22q11.21)	7	0.28
XO	17	0.68	del (17q12)	5	0.20
XXY	17	0.68	dup (7q36.3)	4	0.16
XXX	12	0.48	del (16p13.11)	4	0.16
XYY	12	0.48	dup (17p13.3)	3	0.12
T13	8	0.32	del (Xp21.1)	3	0.12
XXXXY	1	0.04	del (2p16.3)	3	0.12
Total	164	6.52	del (Xp22.31)	3	0.12
			dup (Xq27.1)	3	0.12
			dup (9p24.3)	2	0.08
			dup (22q11.21)	2	0.08
			dup (10q24.31)	2	0.08
			del (Yp11.32)	2	0.08
			del (Xp11)	2	0.08
			del (7q11.23)	2	0.08
			del (Xp22.33)	2	0.08
			del (17p12)	2	0.08
			del (16p11.2)	2	0.08
			other 41 CNVs	36	1.43
			Total	98^a^	3.66

^a^ Six samples have been detected with two pathogenic or likely pathogenic CNVs, and three samples have been detected with one aneuploidy and one pathogenic or likely pathogenic CNV.

### 3.3 Chromosomal abnormality rate among the major ultrasound abnormal samples

**FIGURE 4:**
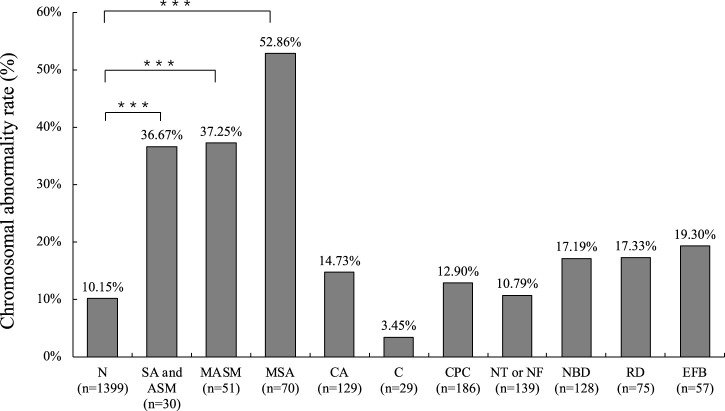
Chromosomal abnormality rate among the major ultrasound abnormal samples. N, No ultrasonic abnormalities. SA and ASM, Structural abnormality and abnormal soft markers. MASA, Multiple abnormal soft markers. MSA, Multiple structural abnormalities. CA, Cardiac abnormalities. C, Chilopalatognathus. CPC, Choroid plexus cyst. NBD, Nasal bone dysplasia. RD, Renal dysplasia. EFB, Echogenic fetal bowel. ***p < 0.001.

Chromosomal abnormalities were identified in 18.39% of fetuses with abnormal ultrasound, which was significantly higher than the percentage found in fetuses with normal ultrasound (*p* < 0.001) (Table 1). Professionals classified the ultrasound diagnosis results for structural or soft markers abnormalities. The ultrasound results indicate that cardiac abnormalities (5.13%, 129/2514) and chilopalatognathus (1.15%, 29/2514) are the most common structural abnormalities. The most common soft marker abnormalities are choroid plexus cyst (7.40%, 186/2514), NT or NF thickening (5.53%, 139/2514), nasal bone dysplasia (5.09%, 128/2514), renal dysplasia (2.98%, 75/2514), and echogenic fetal bowel (2.27%, 57/2514) (Figure 4; Supplementary Table S5). We found that samples with multiple ultrasonic abnormalities had a significantly higher rate of chromosomal abnormalities (*p* < 0.001) compared to normal ultrasonic samples. Single-indicator ultrasonic abnormal samples such as nasal bone dysplasia, renal dysplasia, or echogenic fetal bowel also had a higher rate of chromosomal abnormalities (*p* < 0.05) (Figure 4).

### 3.4 Genetic analysis of VUS CNVs through trio families

Of the fetuses in our cohort, 55.73% were detected with VUS CNVs. To further analyze the genetic origin of these VUS CNVs, we conducted a mining analysis of Trio family data. Out of the 1401 samples with VUS CNVs, 521 samples had Trio family CNV-seq results. A total of 780 CNVs were detected in 521 fetuses, including 41 pathogenic CNVs, 7 likely pathogenic CNVs, 683 VUS CNVs, and 49 benign CNVs (Supplementary Table S6; Table 3). The corresponding inherited rates were 43.90%, 0%, 94.00%, and 87.76%.

**TABLE 3:** Parental verification to investigate origin of copy number variations.

Type of CNV	Fetal CNVs detected (N)	Inherited CNVs (N)	*De novo* CNVs (N)	Inherited rate (%)
Pathogenic	41	18	23	43.90
Likely pathogenic	7	0	7	0
VUS	683	642	41	94.00
Likely benign	49	43	6	87.76

## 4 Discussion

Using an integrated strategy of CNV-seq and karyotype analysis, our study detected chromosomal abnormalities in 13.80% of pregnant women (347/2514), which is consistent with a previous study published by Lan et al., in 2021 (13.47%, 123/913) (Lan et al., 2021). CNV-seq, by adjusting sequencing depth and achieving a resolution of 100 kb, complemented karyotype analysis’s limitations. Among fetuses with normal karyotypes, CNV-Seq diagnosed 66 (2.63%) cases of pathogenic CNVs ranging from 132.32 Kb to 4.60 Mb, which were all below the detection range of karyotyping. This underscores CNV-seq’s enhanced diagnostic capability, corroborated by Wang et al., who observed a detection rate increase from 1.8% to 2.8% for pathogenic or likely pathogenic variants (Wang J. et al., 2018). Furthermore, CNV-seq technology aids karyotyping in precisely localizing chromosomal abnormalities at the genomic. Despite its advantages, CNV-seq faces challenges in discerning CNVs within highly homologous or repetitive regions due to the limitation of NGS platforms. It also fails to identify triploidy, polyploidy, and balanced structural abnormalities, such as translocations or inversions. Among the 94 samples exclusively detected by karyotyping, 76 exhibited translocations or inversions, with CNV-seq results indicating no pathogenic genetic material change, highlighting that structural chromosomal abnormalities do not always portend a poor prognosis. While the integrated approach incurs higher costs than either CNV-seq or karyotyping alone, the decreasing costs of next-generation sequencing have made CNV-seq increasingly accessible, with prices in major Chinese cities stabilizing at approximately 1400 yuan per case, comparable to karyotyping and less than half the cost of SNP-array. This affordability enhances the integrated strategy’s viability for fetuses requiring invasive diagnostic procedures, mitigating the risk of misdiagnosis. The integrated strategy thus offers a comprehensive prenatal genetic assessment, essential for genetic counseling and informing clinical intervention strategies.

Cardiac abnormalities are the most common structural abnormalities in ultrasound abnormalities and are also the major birth defects in newborns (Van Der Linde et al., 2011; Jorgensen et al., 2014; Wang Y. et al., 2018). Chromosomal abnormalities are the earliest confirmed cause of CHD, accounting for about 9%–18% of CHD cases, and 28%–45% of CHD cases diagnosed by a prenatal diagnosis have chromosomal abnormalities (Mademont-Soler et al., 2013). In this study, the incidence of congenital heart disease in the prenatal diagnosis cohort of chromosomal abnormalities was 5.3%. Chromosomal abnormalities were identified in 14.73% of samples. There was no significant difference in the chromosome detection rate of cardiac abnormalities compared to samples without ultrasound abnormalities (*p* = 0.071), but there was a high trend. Consistent with previous studies, this study found that using multiple ultrasound abnormality index samples resulted in a higher detection rate of chromosomal abnormalities (*p* < 0.001) (Hu et al., 2021). The results indicate that abnormalities in nasal bone dysplasia, renal dysplasia, and echogenic fetal bowel have a higher detection rate for chromosomal abnormalities compared to choroid plexus cysts, nuchal translucency (NT) thickening, and nuchal fold (NF) thickening. Furthermore, these abnormalities are statistically significant when compared to samples without ultrasound anomalies. The rate of chromosomal abnormalities is higher among the major ultrasound abnormal samples.

Based on genetic analysis of VUS CNVs in Trio families, we found that 94% of VUS CNVs were inherited from parents, higher than the inherited rate of benign CNVs, indicating that most of them are non-pathogenic variants. These findings could provide important insights and references for correlating prenatal screening indicators with chromosomal abnormalities.

## Data Availability

The datasets presented in this study can be found in online repositories. The raw sequencing data of CNV-seq has been deposited in CNSA database (https://db.cngb.org/cnsa), and the accession number is CNP0005579. The repository is still in the controlled stage of review. After the data review, the data is available from the corresponding author on request.
